# Association of comprehensiveness of antiretroviral care and detectable HIV viral load suppression among pregnant and postpartum women in the Democratic Republic of the Congo: a cross-sectional study

**DOI:** 10.3389/fgwh.2024.1308019

**Published:** 2024-06-05

**Authors:** Alix Boisson-Walsh, Noro L. R. Ravelomanana, Martine Tabala, Fathy Malongo, Bienvenu Kawende, Pélagie Babakazo, Marcel Yotebieng

**Affiliations:** ^1^ Department of Health Policy and Management, University of Carolina at Chapel Hill, Chapel Hill, NC, United States; ^2^ School of Public Health, University of Kinshasa, Kinshasa, Democratic Republic of Congo; ^3^ Division of General Internal Medicine, Department of Medicine, Albert Einstein College of Medicine, Bronx, NY, United States

**Keywords:** antiretroviral therapy, virological suppression, pregnant and breastfeeding women living with HIV, ART service readiness, Democratic Republic of Congo, maternal and child health clinics

## Abstract

**Introduction:**

Worldwide, over two-thirds of people living with HIV are on antiretroviral therapy (ART). Despite increased ART access, high virological suppression prevalence remains out of reach. Few studies consider the quality of ART services and their impact on recipients' viral suppression. We assessed the association between ART service readiness and HIV viral load suppression among pregnant and breastfeeding women living with HIV (WLH) receiving ART in maternal and child health (MCH) clinics in Kinshasa, Democratic Republic of Congo.

**Methods:**

We performed a cross-sectional analysis leveraging data from a continuous quality improvement intervention on WLH's long-term ART outcomes. From November 2016 to May 2020, we enrolled WLH from the three largest clinics in each of Kinshasa'Łs 35 health zones. We measured clinic's readiness using three WHO-identified ART care quality indicators: relevant guidelines in ART service area, stocks of essential ART medicines, and relevant staff training in ≥24 months, scoring clinics 0-3 based on observed indicators. We defined viral load suppression as ≤1,000 cp/ml. Multilevel mixed-effect logistic models were used to estimate prevalence odds ratios (ORs) measuring the strength of the association between ART service readiness and viral suppression.

**Results:**

Of 2,295 WLH, only 1.9% received care from a clinic with a score of 3, 24.1% received care from a 0-scoring clinic, and overall, 66.5% achieved virologically suppression. Suppression increased from 65% among WLH receiving care in 0-scoring clinics to 66.9% in 1-scoring clinics, 65.8% in 2-scoring clinics, and 76.1% in 3-scoring clinics. We did not observe a statistically significant association between ART service readiness score and increased viral suppression prevalence, however we did find associations between other factors, such as the location of the health center and pharmacist availability with suppressed viral load.

**Discussion:**

A lack of comprehensive ART care underscores the need for enhanced structural and organizational support to improve virological suppression and overall health outcomes for women living with HIV..

## 1 Introduction

Despite significant advances in antiretroviral (ART) services contributing to an estimated 12.1 million AIDS-related deaths, (1, 2) achieving the ambitious UNAIDS 90-90-90 targets [90% of people living with HIV (PLHIV) are aware of their status, of whom 90% have initiated ART treatment, and of whom 90% have achieved virologic suppression by 2020] remains a challenge as of December 2022, with considerable disparities across regions and population groups (3–5). Due to an unprecedented initiative to eliminate mother-to-child transmission, pregnant women have generally had better access to HIV testing and ART than the general population during antenatal care visits to maternal and child health (MCH) clinics. However, the provision of longitudinal chronic ART care through maternal and child health (MCH) clinics, designed for episodic acute care, has not been conclusively shown to yield high viral suppression. Furthermore, while pregnant and breastfeeding women living with HIV (WLH) receive treatment, viral suppression remains suboptimal, and significant variability in suppression rates across facilities remains (6).

Existing literature has predominantly focused on individual-level factors affecting virological suppression among pregnant and breastfeeding WLH, (7, 8) with scant attention to the healthcare delivery system's design, which is integral to performance and health outcomes (9). Our study aims to bridge this gap by examining the association between clinic-level ART service quality and the prevalence of virological suppression among pregnant and postpartum WLH in the Kinshasa Province, Democratic Republic of Congo (DRC).

Underpinned by the Systems Theory of Management, (10) which posits that the interrelationships among its subsystems determine an organization's effectiveness, this study scrutinizes the structural and process aspects of ART service delivery within MCH clinics. The theory guides our investigation into how systemic attributes such as service readiness, staff capacity, and clinic management practices influence the ultimate goal of virological suppression. By aligning our research design with this framework, we aim to highlight the complexities of service delivery and provide insights into system-level interventions that can enhance outcomes for WLH in the DRC.

## 2 Methods

### 2.1 Study design, setting, and population

We performed a cross-sectional analysis of data from the baseline enrollment assessment of the continuous quality intervention-prevention-of-mother-to-child transmission (CQI-PMTCT) study: a cluster randomized trial to evaluate the effect of CQIs on long-term ART outcomes among pregnant and breastfeeding women (NCT03048669) (11). The study selected the three clinics with the highest patient volume in each of the 35 health zones of the Kinshasa province in the Democratic Republic of Congo. From June to May 2017, study staff visited each of the 105 MCH clinics and interviewed departmental supervisors or managers to collect clinic-level information using a facility survey tool modeled after a facility audit of service quality developed by MEASURE Evaluation to assess the quality and provision of district-level health services (12). The same study staff completed the entire questionnaire within a facility. From November 2016 to May 2020, pregnant or breastfeeding (≤1-year post-delivery) WLH receiving ART in the clinics were approached for enrollment. Participants were enrolled at any time during antenatal care visits, immediately after delivery (in the maternity ward during the 1–3 days post-delivery observation), or during routine well-child visits in the postpartum period. All participants were informed about the study and provided written consent. The Institutional Review Board of the Ohio State University and the Kinshasa School of Public Health committee approved the protocol. After receiving consent, the study team collected five 50 ml blood spots obtained by a finger prick on Whatman paper from each participant. The samples were sent to the national HIV reference laboratory for viral load measurement.

### 2.2 Ethical considerations

Ethical approval was received from the Institutional Review Boards at Ohio State University and the Kinshasa School of Public Health in the Democratic Republic of the Congo. Participants' written consent to participate in this study was obtained during enrollment. Participants' identification were anonymized in the study data using participant identification codes in the relevant dataset.

### 2.3 Variables

#### 2.3.1 Virological suppression

The primary outcome of this analysis was virological suppression, defined as a viral load of ≤1,000 copies/ml.

#### 2.3.2 ART service readiness

The study's primary exposure was ART service readiness. The ART service readiness variable was calculated for each maternity clinic, using three WHO-identified inputs to quantify service-specific readiness obtained from the facility survey data (13): (1) whether relevant health staff member(s) received ART service-related training within the last 24 months; (2) whether country-specific ART service guidelines were available in the service area; (3) whether first-line ART regimens were available (TDF + 3TC + EFV, TDF + FTC + EFV, AZT + 3TC + EFV, TDF + 3TC + NVP, TDF + FTC + NVP, and AZT + 3TC + NVP). We assigned equal weight to each component in line with the WHO approach. The study team attributed a score of one for each indicator that was observed during the clinic survey. Indicators that were reported but not observed received a score of 0. Each clinic received a final measure of ART service readiness ranging from 0 (no indicator observed) to 3 (all three indicators observed) based on the number of indicators observed.

#### 2.3.3 Other covariables

Covariates at the MCH clinic level included: clinic type (hospital or health center), operational entity (public, private-for-profit, or non-profit), location (urban or peri-urban/rural), support by a PEPFAR implementing partner (yes or no), external supervision provided by the health zone bureau (yes or no), and the number of healthcare staff (continuous). Covariates at the individual level included: the participant's ART regimen (TDF + 3TC + FEV, AZT + 3TC + NVP, or Lopinavir-based), (14–16) her age in years (≤24, 25–34, or 35+), her marital status (married/cohabitating or divorced/separated/widowed/never married), her duration of ART treatment (<12, 12–24, or 24 months), (15, 17–23) her educational attainment (primary, secondary, or tertiary), (16, 17) whether she disclosed her HIV status (yes or no), (17) and her wealth index score. To compute the wealth index score, we employed principal component analysis considering the following factors: years of education, number of household members per room, number of sleeping beds in the household, household water source (communal vs. private pipe), cooking fuel type (electrical stove vs. wood/charcoal), and status of ownership of prosaic household goods (mobile phone, radio, fridge, vehicle, bike, and motorcycle). To replicate previous studies, (6, 24) we retained the first component accounting for the most significant variability in the dataset (20.7%). We categorized the first component into quintiles from lowest to highest SES (0–4) to produce the wealth index score.

### 2.4 Statistical analysis

We calculated the proportion of women with virologic suppression and compared it across levels of ART service readiness and other key variables. We used bivariate multilevel mixed-effect logistic models regressions to estimate prevalence odds ratios (ORs) and 95% confidence intervals (95% CIs), measuring the strength of association between potential confounders and the outcome. We included variables found in bivariate analyses to be associated with viral load suppression (*P* <= 0.20) in the multivariate model. We employed a backward selection methodology to select the most parsimonious final model that minimized bias and optimized the precision of the effect estimate. We retained confounders in the final model if their exclusion changed the estimate (OR) of the association between exposure and outcome by >10%. Each model accounts for clustering at the MCH clinic and the health zone level. We ran the model with the ART readiness indicator, but also with the three individual readiness indicators to assess model sensitivity to exposure definition. We conducted all statistical analyses using Stata Version 15.0 (StataCorp, College Station, TX, USA), and all statistical tests were two-sided with an alpha level of 0.05 except when otherwise indicated.

## 3 Results

### 3.1 Participant characteristics

The parent study enrolled 2,343 WLH, of whom 55 WLH had no viral load results for a total effective sample size of 2,295 WLH. Most participants (62%) were enrolled during pregnancy or at delivery (Table 1). One thousand three hundred ten (57.1%) participants were diagnosed during their past pregnancy. Over half of the participants (52.1%) fell within the 25–34 age category. Most were married or cohabitating (69.3%), had secondary education (71.7%), and were taking tenofovir-lamivudine-efavirenz (78.2%). Most women received HIV care at a hospital (57.1%) funded by PEPFAR (66.7%) in an urban setting (92.7%).

**Table 1:** Characteristics of pregnant and breastfeeding women, enrolled in a trial evaluating the effect of data-driven continuous quality improvement on long-term ART outcomes in Kinshasa, DRC.

Baseline characteristics	Overall	No indicators present at clinic	1 indicator present at clinic	2 indicators present at clinic	All indicators present at clinic
	2,295 (100)	552 (24.1)	1,326 (57.8)	371 (16.2)	46 (2.0)
	*N* (%)	*N* (%)	*N* (%)	*N* (%)	*N* (%)
Moment of viral load testing					
After pregnancy	873 (38.0)	223 (40.4)	490 (37.0)	143 (38.5)	17 (37)
Pregnancy	1,422 (62.0)	329 (59.6)	836 (63.1)	228 (61.5)	29 (63)
Timing of diagnosis					
Current pregnancy	983 (42.8)	229 (41.5)	562 (42.4)	175 (47.2)	17 (37)
Past pregnancy	1,310 (57.1)	322 (58.3)	763 (57.5)	196 (52.8)	29 (63)
Missing	2 (0.1)	1 (0.2)	1 (0.1)	0 (0)	0 (0)
Marital status					
Other	703 (30.6)	174 (31.5)	428 (32.3)	95 (25.6)	6 (13)
Married/cohabitating	1,589 (69.3)	377 (68.3)	896 (67.6)	276 (74.4)	40 (87)
Missing	3 (0.1)	1 (0.2)	2 (0.2)	0 (0)	0 (0)
Age					
<=24	387 (16.5)	96 (17.4)	214 (16.1)	65 (17.5)	3 (6.5)
25–34	1,196 (52.1)	284 (51.4)	693 (51.5)	204 (55.0)	25 (54.3)
35+	719 (31.3)	172 (31.3)	427 (32.2)	102 (275)	18 (39.1)
Missing	2 (0.1)	0 (0)	2 (0.1)	0 (0)	0 (0)
Disclosure of HIV status					
No	1,070 (46.6)	239 (43.3)	638 (48.1)	181 (48.8)	12 (26.1)
Yes	1,220 (53.2)	311 (56.3)	685 (51.7)	190 (51.2)	34 (73.9)
Missing	5 (0.2)	2 (0.4)	3 (0.2)	0 (0)	0 (0)
Primigravida					
Yes	253 (11.0)	65 (11.8)	136 (10.3)	45 (12.1)	7 (15.2)
No	2,041 (88.9)	487 (88.2)	1,189 (89.7)	326 (87.9)	39 (84.8)
Missing	1 (0.0)	0 (0)	1 (0.1)	0 (0)	0 (0)
Educational level					
Primary	275 (12)	65 (11.8)	166 (12.5)	41 (11.1)	3 (6.5)
Secondary	1,646 (71.7)	392 (71.0)	966 (72.9)	250 (67.4)	38 (82.6)
Tertiary	368 (16.0)	94 (17.0)	190 (14.3)	79 (21.3)	5 (10.9)
Missing	6 (0.3)	1 (0.2)	4 (0.3)	1 (0.3)	0 (0)
ART regimen					
TDF + 3TC + FEV	1,794 (78.2)	440 (79.6)	1,032 (77.8)	289 (77.9)	33 (71.7)
AZT + 3TC + NVP	243 (12.0)	66 (12.0)	132 (10.0)	34 (9.2)	11 (23.9)
Lopinavir-based	251 (7.8)	43 (7.8)	158 (11.9)	48 (12.9)	2 (4.3)
Other	7 (0.5)	3 (0.5)	4 (0.3)	0 (0)	0 (0)
Wealth index in quartiles					
1 (poorest)	561 (24.4)	158 (28.6)	304 (22.9)	89 (24.0)	10 (21.7)
2	571 (24.9)	121 (22.0)	347 (26.2)	91 (24.5)	12 (26.1)
3	574 (25.0)	152 (27.6)	328 (24.8)	84 (21.8)	10 (21.7)
4 (least poor)	598 (25.7)	121 (22.0)	347 (26.2)	107 (28.8)	14 (30.4)
Appropriation					
Private for profit	264 (11.5)	56 (10.1)	145 (11.0)	63 (17.0)	0 (0)
Non-profit	1,318 (57.4)	208 (37.7)	792 (59.7)	272 (73.3)	46 (100)
Government/public	713 (31.1)	288 (52.2)	389 (29.3)	36 (9.7)	0 (0)
Clinic type					
Health center	984 (42.9)	210 (38.0)	477 (36.0)	271 (73.1)	26 (56.5)
Hospital	1,311 (57.1)	342 (61.2)	849 (64.0)	100 (27.0)	20 (43.5)
PEPFAR funding					
No	765 (33.3)	249 (45.1)	390 (29.4)	126 (34.0)	0 (0)
Yes	1,530 (66.7)	303 (54.9)	936 (71.0)	245 (66.0)	46 (100)
Clinic location					
Peri-urban/rural	166 (7.2)	69 (12.5)	62 (4.7)	23 (6.2)	12 (26.1)
Urban	2,129 (92.7)	483 (87.5)	1,264 (95.3)	348 (93.8)	34 (73.9)
External supervision					
>6 months	128 (5.6)	29 (5.3)	57 (4.3)	42 (11.3)	0 (0)
≤6 months	2,167 (94.4)	523 (94.8)	1,269 (95.7)	329 (88.7)	46 (100)
Pharmacist					
Not available	1,476 (64.3)	262 (47.5)	839 (63.3)	329 (88.7)	46 (100)
Available	819 (35.7)	290 (52.5)	487 (36.7)	42 (11.3)	0 (0)

### 3.2 MCH clinic characteristics

Of the 99 facilities participating in the survey assessment, only 5 (5.1%) had an ART services readiness score of 3, 16 (16.2%) had a score of 2, 48 (48.5%) had a score of, and 30 (30.3%) had a score of 0. Among the facilities, 64 (64.7%) had country-specific ART service guidelines available in the service area, 19 (19.2%) provided ART service-related training, and 13 (13.1%) had first-line ART regimens available. The slight majority of health facilities were hospitals (52.5%), were located in urban settings (85.9%), were managed by a not-for-profit (44.4% vs. 23.2% private and 32.3% not-for-profit), were supported by a PEPFAR implementing partner (51.5%) and did receive external supervision by the health zone bureau within the last 6 months (96%) (Table 2). Most facilities (81.8%) did not have a pharmacist available. Of the 2,295 WLH included in the analysis, 24.1% (552) received care from a clinic with an ART readiness score of 0, 57.8% (1,326) from a clinic with a score of 1, 16.2%, (371) from a 2-scoring clinic, and only 2% (46) received care from a clinic with an ART readiness score of 3.

**Table 2:** Site characteristics; Kinshasa health facilities survey, 2016.

Facility characteristics	Overall		No indicators present		1 indicator present		2 indicators present		All indicators present	
	(*n* = 99)		(*n* = 30)		(*n* = 48)		(*n* = 16)		(*n* = 5)	
	*N*	(%)	*N*	(%)	*N*	(%)	*N*	(%)	*N*	(%)
Appropriation										
Private for-profit	23	(23)	8	(26)	11	(23)	4	(25)	0	(0)
Not-for-profit	44	(44)	11	(36)	19	(38)	9	(56)	5	(100)
Government/public	32	(32)	11	(36)	18	(38)	3	(19)	0	(0)
Type of facility										
Health center	47	(47)	16	(53)	19	(40)	9	(56)	3	(60)
Hospital	52	(53)	14	(47)	29	(60)	7	(44)	2	(40)
PEPFAR funding										
No	48	(48)	18	(60)	24	(50)	6	(38)	0	(0)
Yes	51	(52)	12	(40)	24	(50)	10	(62)	5	(100)
Location										
Peri-urban/rural	14	(14)	5	(17)	6	(75)	2	(13)	1	(20)
Urban	85	(86)	25	(83)	42	(25)	14	(87)	4	(80)
External supervision										
>6 months	4	(4)	2	(7)	1	(2)	1	(6)	0	(0)
≤6 months	95	(96)	28	(93)	47	(98)	15	(94)	5	(100)
Pharmacists										
Available	18	(18)	7	(23)	10	(21)	1	(6)	0	(0)
Not available	81	(82)	23	(77)	38	(79)	15	(94)	5	(100)

PEPFAR, president's emergency plan for AIDS relief.

### 3.3 Bivariate analysis

From the analytical sample of 2,295 women, 66.5% (1,525) were virologically suppressed (Table 3).

**Table 3:** Relationship between readiness of ART service and suppressed HIV viral load.

	Suppressed VL *n*/*N* (%)	uOR (95% CI)	aOR (95% CI)	Ind1OR (95% CI)	Ind2OR (95% CI)	Ind3OR (95% CI)
	1,525/2,379 (66.5)					
ART service readiness						
0 (poor)	359/552 (65.0)	1	1	–	–	–
1	887/1,326 (66.9)	1.05 (0.80, 1.38)	1.04 (0.84, 1.30)	–	–	–
2	244/371 (65.8)	1.11 (0.74, 1.64)	1.07 (0.79, 1.45)	–	–	–
3 (comprehensive)	35/46 (76.1)	1.82 (0.84, 3.94)	1.79 (0.87, 3.68)	–	–	–
Training received by staff						
No		–	–	1	–	–
Yes		–	–	1.09 (0.85, 1.40)	–	–
National guidelines						
No		–	–	–	1	–
Yes		–	–	–	1.15 (0.94, 1.43)	–
Availability of first-line ART						
No		–	–	–	–	1
Yes		–	–	–	–	0.92 (0.66, 1.29)
Appropriation						
Private for profit		1	1	1	1	1
Non-profit		1.25 (0.91, 1.72)	1.23 (0.93, 1.64)	1.25 (0.94, 1.67)	1.23 (0.93, 1.65)	1.25 (0.94, 1.67)
Public		1.10 (0.91, 1.72)	1.02 (0.74, 1.41)	1.02 (074, 1.41)	1.04 (0.75, 1.43)	1.02 (0.74, 1.41)
Facility type						
Health center		1	1	1	1	1
Hospital		1.07 (0.85, 1.35)	0.98 (0.80, 1.19)	0.99 (0.81,1.20)	0.97 (0.79, 1.18)	0.97 (0.80, 1.18)
PEPFAR funding						
No		1	1	1	1	1
Yes		1.20 (093, 1.53)	1.07 (0.87, 1.31)	1.08 (0.88,1.32)	1.08 (0.87, 1.33)	1.09 (0.89, 1.33)
Location						
Peri-urban/rural		1	1	1	1	1
Urban		1.58 (1.09, 2.30)	1.56 (1.11, 2.20)	1.55 (1.11, 2.16)	1.48 (1.05, 2.08)	1.54 (1.10, 2.15)
External supervision						
>6 months		1	1	1	1	1
≤6 months		1.51 (0.94, 2.41)	1.54 (1.08, 1.55)	1.55 (1.11, 2.16)	1.56 (1.04, 2.35)	1.30 (1.03, 2.31)
Pharmacists						
Not available		1	1	1	1	1
Available		1.28 (1.01, 1.62)	1.34 (0.36, 1.14)	1.32 (1.07, 1.63)	1.34(1.08, 1.67)	0.68(0.39, 1.19)

VL, viral load; uOR, unadjusted odds ratio; aOR, adjusted odds ratio.

### 3.4 Association of comprehensiveness of ART service with suppressed viral load

The prevalence of virologic suppression was 65.0% among participants receiving care in a clinic with an ART readiness score of 0 compared to 66.9% among those receiving care in a 1-scoring clinic (cOR: 1.05, 95% CI: 0.80, 1.38), or 65.8% in those in 2-scoring clinics (cOR: 1.11, 95% CI: 0.74, 1.64), and 76.1% in 3-scoring clinics (cOR: 1.82, 95% CI: 0.84, 3.94). After adjusting for the operational entity, type of clinic, PEPFAR funding, routine quality assurance activities, external supervision of the clinic, and the pharmacists' availability at the health clinic, and study phase, the prevalence ratios estimate remain unchanged [aOR1: 1.04, 95% CI: 0.84, 1.38; aOR2: 1.07, 95% CI: 0.79, 1.45; aOR3: 1.79, 95% CI: 0.87, 3.68, respectively, (Table 3)]. Further, individual components of the ART readiness indicator did not yield statistically significant estimates (refer to Table 3, models Ind1ME, Ind2ME, and Ind3ME).

### 3.5 Association of covariates with suppressed viral load

In our investigation of factors correlated with suppressed viral load among HIV-positive individuals, the primary exposure variable, ART service readiness, did not show a statistically significant impact. However, our analysis identified two key covariates consistently associated with an increased likelihood of viral suppression. Patients treated in urban health centers had 1.58 times the odds of achieving viral suppression compared to those in peri-urban centers (95% CI: 1.09, 2.30). Patients also had 1.28 times the odds of achieving suppressed viral load if the health facility had a pharmacist available (95% CI: 1.01, 1.62). In addition, each model evaluating individual exposure variables and the adjusted model showed that the experience of external supervision within the past 6 months contributed positively to suppressed viral load.

## 4 Discussion

Among our sample of WLHs who had their first viral load collected, only 1.9% received care in a clinic with comprehensive ART services, despite every health facility reporting provision and access to antiretroviral (ART) services during the study team visits to the facilities. Our comprehensive analysis of the association between ART service readiness and virological suppression in MCH clinics revealed that, although the primary exposure variable of ART service readiness did not show a statistically significant impact, clinic location and pharmacist availability were critical in enhancing the likelihood of viral suppression. This aligns with previous findings that emphasize the importance of healthcare accessibility and professional support in managing chronic conditions like HIV.

Importantly, our study introduces novel insights into the role of systemic factors, particularly the impact of external supervision, in promoting positive health outcomes. The 44 percentage point increase in viral suppression probability due to such supervision suggests that oversight may catalyze improving service delivery quality, thus enhancing patient outcomes.

Despite these substantial findings, the lack of statistically significant associations in specific areas underlines the complex interplay of factors influencing virological outcomes and the necessity for multifaceted approaches to bolster the ART service framework within MCH clinics.

Among our sample, a higher percentage of participants seeking care at facilities with comprehensive ART service readiness scores (76.1%) achieved viral suppression compared to those seeking care at 0-scoring facilities (65%), however this finding is not statistically significant. Likewise, WLH seeking care in facilities with one or two ART readiness indicators were more likely to have a suppressed HIV viral load detected than those seeking care at facilities with no readiness indicators observed. Although these findings were not statistically significant, they highlight the importance of expanding the availability of core PMTCT interventions and strengthening the capacity of facilities and relevant personnel to offer these interventions.

Several possibilities might explain the increased viral load suppression among higher-scoring ART service readiness facilities at the organizational level. Unsupported staff may not possess the tools and training required to appropriately educate women on the risk of MTCT, (25) which also may impact adherence to ART treatment. Poorly trained healthcare staff may not have the necessary resources and training to counsel WLH, which could also act as a determinant of HIV viral load. Without clinical directives, WLH may fail to report their medical status to their sexual partners (26, 27). Further, a health system's fragmentation may deepen in cases where communication of updated guidelines and protocols related to ART service areas do not reach healthcare managers and staff (28). The resulting impact on quality of care, lack of organization, and inefficiency of services at an organizational level could lead to overcrowding of health facilities and long treatment and service delays (29–31). The limitations may severely deter a facility's attendance. We speculate maternal disengagement from the ART cascade may mediate the association between ART service readiness and viral load. A previous study found an association between quality improvement activities and WLH retention in public-sector clinics (32).

Stockouts of HIV commodities, such as ART, represent a structural inadequacy that broadly impacts PMTCT programs (33). International organizations, such as PEPFAR, offer logistical and technical support to alleviate structural barriers in low-resource settings, such as the DRC (34). However, decision-makers in health systems receiving such funds should conduct system-based monitoring to forecast approaches to provision for future international funding reductions (35, 36) This proves especially true given the inadequacies of DRC's public healthcare spending, which accounts for only 9% of its budget (37).

The interpretation of our findings should account for several limitations. First, departmental supervisors and managers reported survey data. As such, we could not preclude over or under-estimation of ART care readiness. We decreased the possibility of differential misclassification through regular surveys with departmental supervisors and managers instead of a composition of staff members. Another limitation was that our estimates account only for women receiving care in our study clinics. Therefore, we cannot adequately estimate the burden of HIV viral load among WLH seeking care in other MCH clinics or those not seeking clinical care. Dried blood spot viral load systematically underestimates plasma viral load (38, 39). We, therefore, undoubtedly underestimated the actual proportion of women without suppressed viral loads. Another limitation is that our data lack information on adherence between ART initiation and date of enrollment. We, therefore, cannot assess whether adherence is a mediator in the observed association between ART comprehensiveness and viral load suppression. In addition, the parent study recruited participants from the three MCH clinics with the highest patient volume in each of the 35 health zones in Kinshasa Province. Data from these clinics may not represent trends in other DRC-based MCH clinics. However, due to elevated patient rates, findings from the 105 clinics most likely reflect WLH receiving care in Kinshasa province. Finally, considering that the study involved high-volume clinics in Kinshasa, the results may suggest wider trends in ART service effectiveness and patient outcomes throughout the province, especially in resource-rich urban areas. Nevertheless, given the variability in clinic operations and resources, careful consideration is needed when applying these findings to all Kinshasa facilities. Further studies are warranted to explore how these findings might be scaled across the province's diverse healthcare settings

Our study found that, among the 99 MCH clinics in Kinshasa Province, only four had all three ART readiness indicators present, and 98.1% of WLH did not receive care in a facility with comprehensive ART care. Despite the non-statistically significant results, the trend suggests that comprehensive ART services correlate with better virological outcomes. Addressing the barriers to quality ART services is imperative for MCH clinics to enhance the care outcomes for WLH, particularly in achieving viral suppression. As we continue to unravel the complexities of healthcare delivery and its impact on HIV management, it is evident that robust structural and organizational support systems are essential. Our findings call for focused interventions that support WLH and strengthen the healthcare infrastructure catering to their needs. Through such concerted efforts, we can see a marked improvement in the health outcomes for WLH in Kinshasa and across similar settings.

## Data Availability

The original contributions presented in the study are included in the article/Supplementary Material, further inquiries can be directed to the corresponding author.
